# Pharmacokinetics, Withdrawal Time, and Physiological Effects of Single Oral Administration of Enrofloxacin in Dybowski’s Frog (*Rana dybowskii*)

**DOI:** 10.3390/antibiotics14040417

**Published:** 2025-04-19

**Authors:** Yanan Wang, Jing Wang, Ran Zhao, Shaowu Li, Guo Hu, Di Wang

**Affiliations:** 1 Key Laboratory of Aquatic Animal Diseases and Immune Technology of Heilongjiang Province, Heilongjiang River Fisheries Research Institute, Chinese Academy of Fishery Sciences, Harbin 150070, China; 2 College of Fisheries and Life Science, Dalian Ocean University, Dalian 116023, China

**Keywords:** *Rana dybowskii*, enrofloxacin, ciprofloxacin, pharmacokinetic, withdrawal time

## Abstract

**Background**: As a broad-spectrum fluoroquinolone, enrofloxacin (ENR) is commonly employed to manage bacterial infections in aquatic species. Nevertheless, there have been no documented pharmacokinetic and residue studies conducted on Dybowski’s frog (*Rana dybowskii*). Therefore, the objective of our study was to characterize the pharmacokinetics (PK) of ENR and its metabolite ciprofloxacin (CIP) in *R. dybowskii*, establish withdrawal times, and evaluate the physiological effects associated with ENR administration. **Methods**: Adult *Rana dybowskii* (120 individuals; 60 males and 60 females) were sex-separated and acclimated in four tanks. Prior to dosing, three males and three females were randomly selected as untreated controls (without ENR administration). Following the oral gavage of ENR (10 mg/kg), blood, liver, and kidney tissues were collected at 0.25, 0.5, 1, 1.5, 2, 4, 6, 8, 12, 24, 36, 48, and 72 h (n = 6) for pharmacokinetic analysis. Muscle and oviduct tissues were additionally sampled at 1, 3, 7, 15, and 30 days post-dose (n = 6) for ENR content determination. Serum/tissue ENR concentrations were measured via Liquid Chromatography–Tandem Mass Spectrometry (LC-MS/MS) and analyzed using a non-compartmental model (WinNonLin 6.1 software) to calculate PK parameters including peak time (T_max_), peak concentration (C_max_), and area under the curve (AUC_0−t_). In studying the physiology effects of ENR administration, biochemical enzyme activities and gene expressions in the liver and intestine were assessed post-ENR administration. **Results**: ENR demonstrated rapid absorption and extensive distribution in *R. dybowskii*. The withdrawal periods were determined to be over 33 days for females and 34 days for males in *R. dybowskii*. Following ENR administration, there was an increase in immune enzymes (AKP (alkaline phosphatase) and ACP (acid phosphatase)) as well as glycolytic enzymes (HK (hexokinase), PK (pyruvate kinase), PFK (phosphofructokinase)). Antioxidant enzyme levels, specifically SOD (superoxide dismutase) and CAT (catalase), peaked at 1.5 h post-ENR administration but subsequently declined by the 8 h mark. Additionally, following ENR treatment, *IGF1*, *PI3K*, and *Akt* exhibited up-regulation, whereas *Keap1* and *GYS1* showed down-regulation. **Conclusions**: The administration of ENR at a dosage of 10 mg/kg significantly enhances the activities of AKP and ACP, promotes glycolysis, and activates the Keap1/Nrf2 and PI3K-Akt signaling pathways in *R. dybowskii*. These findings establish a foundation for the rational application of ENR and the determination of withdrawal times in frog aquaculture.

## 1. Introduction

As an endangered species of tailless amphibian, Dybowski’s frog (*Rana dybowskii*) belongs to the family Ranidae and the genus *Rana*, which is mainly found in northeast China (Heilongjiang, Jilin, Liaoning, and Inner Mongolia), the Russian Federation, Mongolia, and the Tsushima Island in Japan [1]. The dried oviduct of the female *R. dybowskii*, known as Oviductus Ranae, possesses medicinal properties that are well documented in numerous traditional Chinese pharmaceutical texts [2]. In recent years, due to limited resources and increasing demand, *R. dybowskii* has become consistently scarce and expensive. Throughout the 20th century, the intensive commercial cultivation of *R. dybowskii* emerged to meet the rising needs of consumers. Technological advancements and industry requirements have spurred significant growth in the aquaculture sector for *Rana dybowskii*. However, high-density aquaculture practices coupled with inadequate management have severely constrained industry development due to bacterial diseases [3]. As a result, the prevalence of bacterial infections has surfaced as a major challenge in *R. dybowskii* farming. Red leg syndrome [4] and rotten skin disease [5] are among the most common bacterial ailments affecting amphibians, leading to substantial economic losses within *R. dybowskii* farming operations.

Owing to the absence of vaccines, the use of antibiotics was the most common and effective therapeutic method [6]. Therapy with antibiotics, indispensable in medicine to save lives and minimize postoperative infections, controls bacterial infections by killing bacteria effectively and curbing disease progression and spread [7]. Improper antibiotic use negatively impacts both animal health and human food safety. The residues of veterinary pharmaceuticals in food can pose hazards to human health, such as direct toxic effects and enhanced bacterial resistance [8]. To safeguard human health, antimicrobial residues in food are not permitted to exceed the maximum residue limit (MRL) values [9]. Antibiotics have been broadly utilized to prevent or treat some diseases in aquatic animals for more than 50 years [10]. Of these, enrofloxacin (ENR) is a third-generation fluoroquinolone antibiotic widely used in aquaculture for its excellent pharmacokinetic properties and broad-spectrum antibacterial activity [11]. ENR forms a stable interaction with bacterial DNA gyrase subunit A, thereby impairing the enzyme’s cleavage and ligation, inhibiting bacterial DNA replication, and exhibiting antibacterial activity [12]. ENR is partially deethylated to form ciprofloxacin (CIP), an active metabolite against diverse Gram-negative aerobes, Gram-positive bacteria, mycoplasma, and rickettsial microorganisms in various mammalian and non-mammalian species [13]. So, it has been licensed for its excellent characteristics of safety, efficiency, and broad-spectrum antibacterial properties in many countries’ aquaculture, including China, Vietnam, and South Korea [14]. Numerous countries have set maximum residue limits (MRLs) for ENR. And in China, the MRL for ENR in muscle tissue is set at 100 μg/kg [15]. Therefore, it is crucial to grasp ENR’s unique pharmacokinetics in Dybowski ‘s frogs and set an appropriate withdrawal time to avoid harmful residues in edible tissues.

The pharmacokinetics of enrofloxacin (ENR) exhibit variability across different species, including brown trout (*Salmo trutta fario*) [16], snakehead (*Channa argus*) [17], and tilapia (*Oreochromis niloticus*) [18]. While the effects of ENR on various organisms have been extensively studied, research focusing on amphibians remains limited [19]. There is a notable lack of information regarding antimicrobial administration and its associated pharmacokinetic properties in Dybowski’s frog. To date, no studies have documented the pharmacokinetics and residue aspects of ENR in *R. dybowskii*. Therefore, investigating the pharmacokinetic characteristics of ENR in *R. dybowskii* is of significant scientific importance. In this study, we will examine the pharmacokinetics and tissue distribution patterns of ENR along with its primary metabolite CIP following single oral administration in *R. dybowskii*. This investigation aims to inform clinical medication practices and explore potential effects resulting from ENR administration in Dybowski’s frogs. The objective of this research is to characterize the pharmacokinetic profiles and physiological impacts of enrofloxacin in *Rana dybowskii*, thereby providing a scientific foundation for determining withdrawal times and promoting rational drug use within *R. dybowskii* farming practices.

## 2. Results

### 2.1. Method Verification

**Figure 1:**
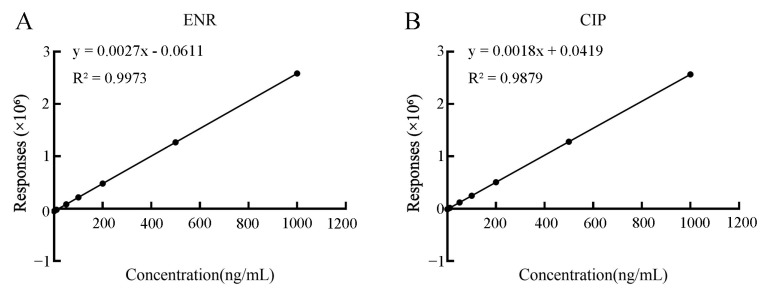
Standard curves for ENR (**A**) and CIP (**B**): linear fitting relationships of responses (×10^6^) with concentration (ng/mL), where the fitting equation for ENR is y = 0.0027x − 0.0611 (R^2^ = 0.9973) and for CIP is y = 0.0018x + 0.0419 (R^2^ = 0.9879).

The detection limit (LOD) and quantitation limit (LOQ) for ENR were detected to be 3 and 1 ng/mL, and for CIP, they were detected to be 0.3 and 1 ng/mL, respectively. As shown in Figure 1, the LC-MS/MS standard calibration curves for ENR and CIP demonstrated linearity within the concentration interval of 1–1200 ng/mL, with correlation coefficients (R^2^) of at least 0.98.

### 2.2. Pharmacokinetics and Elimination of ENR in R. dybowskii

**Figure 2:**
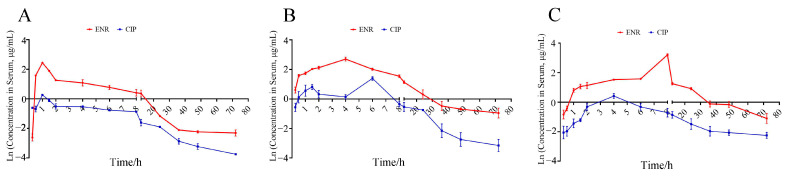
Concentration versus time curve of enrofloxacin (ENR) and ciprofloxacin (CIP) in serum (**A**), liver (**B**), and kidney (**C**) after a single oral dose of ENR (10 mg/kg, n = 6, mean ± SD).

The concentration–time relationships of ENR and CIP in the serum and tissues of *R. dybowskii* are shown in Figure 2A–C, respectively. The PK parameters of ENR following oral administration as a single dose of 10 mg/kg are presented in Table 1. The T_1/2λz_ values of ENR were 12.76 h (serum), 16.77 h (liver), and 17.90 h (kidney), while 28.42 h (serum), 13.18 h (liver), and 81.79 h (kidney) for CIP. The AUC_0−t_ of ENR in serum, liver, and kidney were 65.04 μg/mL⋅h, 141.21, and 142.81 μg/g⋅h, and for CIP, AUC_0−t_ were 10.10 μg/mL⋅h, 32.69, and 18.74 μg/g⋅h.

**Figure 3:**
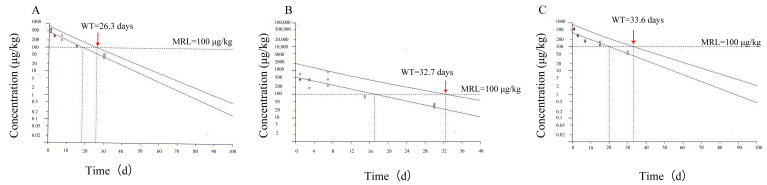
Estimated withdrawal times for enrofloxacin in Dybowski’s frog (*Rana dybowskii*) after oral administrations at 10 mg/kg using the WT 1.4 software. (**A**) for fallopian tubes in female frogs, (**B**) for muscles in female frogs, (**C**) for muscles in male frogs. The two depicted lines in the figure denote the attenuation curves of enrofloxacin in *Rana dybowskii*. The line intersected with the MRL dashed line corresponds to the upper-limit curve with a 95% confidence interval, and the intersection point signifies the withdrawal time (WT).

Log-transformed muscle and oviduct tissue concentration data were regressed linearly. The withdrawal period is defined as the time when the 95% upper tolerance limit drops below an MRL of 100 µg/kg with 95% confidence. As shown in Figure 3, the withdrawal times of the fallopian tubes and muscle were determined as 26.3 and 32.7 days in female frogs and 33.6 days in male frogs.

### 2.3. Biochemical Parameter Alterations in R. dybowskii

**Figure 4:**
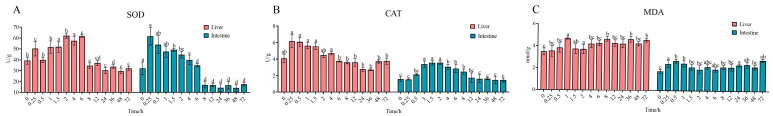
Effects of antioxidant enzyme activity in liver and intestine. (**A**) SOD activity; (**B**) MDA content; (**C**) CAT activity. Values labeled as distinct letters (a–c) denote statistically significant differences (*p* < 0.05).

The dynamics of antioxidant enzyme activities in the liver and intestinal tissues are shown in Figure 4. Compared with pre-ENR administration, SOD and CAT activities exhibited transient increases followed by decreases, whereas malondialdehyde (MDA) content showed a sustained upward trend. Specifically, SOD activity increased significantly (*p* < 0.05) at 4 h after ENR administration, peaking at 62.198 U/g—1.24-fold higher than pre-ENR administration; CAT activity transiently peaked at 0.25 h post-ENR administration and declined by 4 h; MDA content reached a maximum of 4.71 nmol/g at 1 h post-ENR, representing a significant 1.24-fold increase relative to pre-ENR administration levels (*p* < 0.05). In intestinal tissues, SOD and MDA trends mirrored those in the liver, with SOD activity dropping to 14.21 U/g at 48 h post-ENR administration; SOD peaked at 0.25 h post-ENR (1.91-fold than before ENR administration increase) and CAT reached its maximum at 1.5 h post-ENR (2.27-fold higher than before ENR administration).

**Figure 5:**
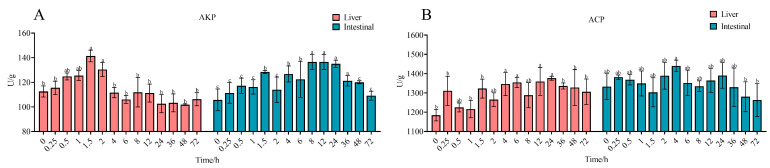
Effects of immune-related enzyme activity in liver and intestine. (**A**) AKP activity; (**B**) ACP activity. Values labeled as distinct letters (a–c) denote statistically significant differences (*p* < 0.05).

Non-specific immune parameters in liver and intestinal tissues are presented in Figure 5. In the liver, compared to pre-ENR administration levels, ACP activity exhibited a sustained upward trend, whereas AKP activity displayed an increase followed by a decline. Specifically, AKP activity peaked at 141.43 U/g at 1.5 h post-ENR administration, representing a 1.25-fold increase over baseline, while ACP activity reached its maximum at 24 h post-ENR administration, measuring 1.16-fold higher than pre-ENR administration values. In intestinal tissues, AKP activity showed a consistent upward trend, with significant elevation (*p* < 0.05) between 8 and 36 h post-ENR administration and a peak of 136.64 U/g at 12 h. Conversely, ACP activity decreased significantly (*p* < 0.05) after 48 h post-ENR administration, reaching a minimum value of 0.96-fold higher than pre-ENR administration levels.

**Figure 6:**
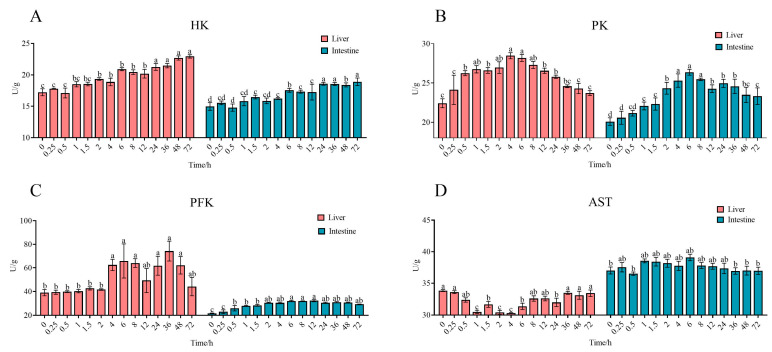
Effects of glucose metabolism in liver and intestine. (**A**) HK activity; (**B**) PK activity; (**C**) PFK activity; (**D**) AST activity. Values labeled as distinct letters (a–d) denote statistically significant differences (*p* < 0.05).

The dynamics of four metabolism-associated enzyme activities are depicted in Figure 6. Following the oral administration of ENR, liver PK and HK activities exhibited upward trends. PFK activity in the liver rose significantly at the 4 h time point, with PK reaching a peak of 28.47 U/g at this interval. HK activity demonstrated a statistically significant increase (*p* < 0.05), culminating in a maximum at 72 h post-ENR administration. PFK activity was notably elevated between 4–8 h and 24–48 h, peaking at 36 h after ENR administration. In intestinal tissues, the three enzymes (PK, HK, PFK) displayed congruent patterns with liver changes. Specifically, intestinal PK activity peaked at 26.36 U/g at 6 h, with a discernible increase observed as early as 1 h post-administration. HK activity reached its peak at 72 h, registering a maximum value of 18.895 U/g. Conversely, PFK activity in the intestine peaked at 12 h, measuring 1.49-fold higher than before ENR administration.

### 2.4. Expression of Keap1/Nrf2 and PI3K-Akt Signaling Pathway

**Figure 7:**
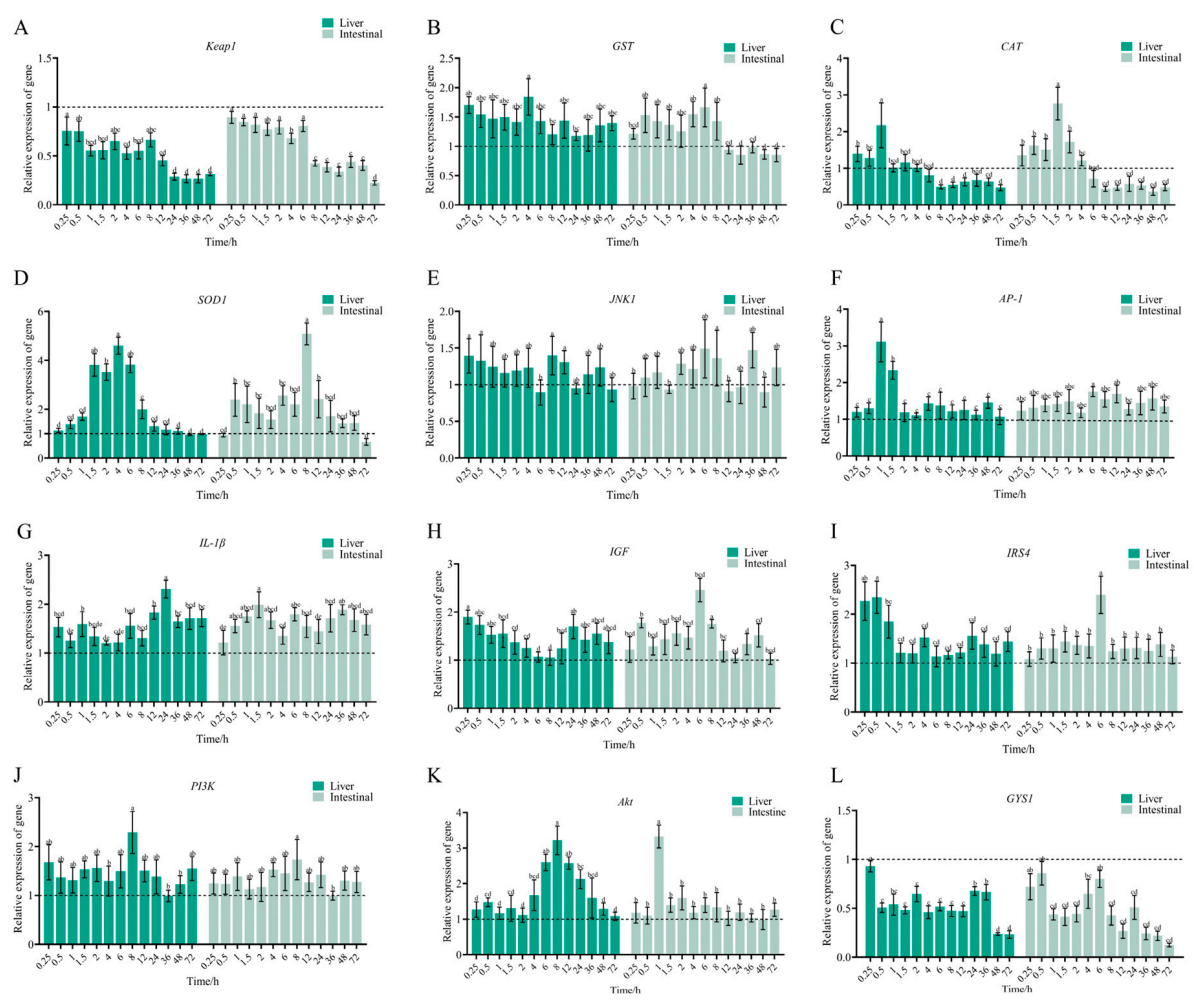
Effects of ENR administration on relative gene expression in the Keap1/Nrf2 signaling pathway (*Keap1* (**A**), *GST* (**B**), *CAT* (**C**), *SOD1* (**D**), *JNK1* (**E**), *AP-1* (**F**), *IL-1β* (**G**)) and the PI3K-Akt signaling pathway (*IGF* (**H**), *IRS4* (**I**), *PI3K* (**J**), *Akt* (**K**), *GYS1* (**L**)) in liver and intestine; Results are presented as mean ± SEM. Values labeled as distinct letters (a-e) denote statistically significant differences (*p* < 0.05).

Gene expression changes in the Keap1/Nrf2 pathway (*Keap1*, *GST*, *SOD1*, *CAT*, *JNK*, *AP-1*, *IL-1β*) are presented in Figure 7. In the liver, *SOD1* and *CAT* expressions were initially up-regulated before declining, whereas *AP-1*, *GST*, and *IL-1β* showed sustained up-regulation, and *Keap1* expression was down-regulated. *JNK1* was down-regulated at 6, 24, and 72 h post-administration but up-regulated at all other time points. *SOD1* expression peaked at 4 h after dosing, reaching 4.6-fold higher than pre-ENR administration levels, before decreasing at 48 h. *CAT* expression peaked at 1 h with a 2.17-fold increase relative to the pre-ENR administration, followed by down-regulation after 6 h. *AP-1* levels peaked at 1 h post-dose, reaching 3.1-fold above pre-ENR administration, while *GST* was up-regulated, peaking at 1.84-fold at 4 h. *Keap1* expression decreased following administration, reaching a nadir of 0.26-fold relative to pre-ENR administration at 36 h. In the intestine, *SOD1*, *CAT*, *AP-1*, *Keap1*, and *IL-1β* exhibited expression patterns analogous to the liver, whereas *GST* showed transient up-regulation followed by down-regulation. *SOD1* was up-regulated as early as 0.5 h before declining, reaching a low of 0.66-fold relative to pre-ENR administration at 72 h. *CAT* expression increased, peaking at 2.76-fold at 1.5 h post-dose. *AP-1* was up-regulated after dosing, reaching a maximum of 1.73-fold at 1.73 h, while *Keap1* expression decreased, with the lowest level (0.22-fold relative to pre-ENR administration) observed at 72 h.

Gene expression trends in the liver and intestine were consistent, as shown in Figure 7. *IRS4*, *PI3K*, and *Akt* were up-regulated, whereas *GYS1* had reduced expression. In the liver, *IRS4* peaked at 0.5 h post-administration, reaching 2.34-fold of the pre-ENR administration levels, while *PI3K* peaked at 8 h, 2.28-fold of the pre-ENR administration levels. *Akt* expression peaked at 8 h post-ENR administration, 3.21-fold higher than pre-ENR administration levels. *GYS1* expression decreased following administration, dropping to 0.23-fold of the pre-administration levels at 72 h. In the intestine, *IRS4* was up-regulated after dosing, peaking at 6 h post-administration (2.39-fold of the pre-ENR administration levels); *PI3K* peaked at 8 h (1.73-fold of the pre-ENR administration levels); and *Akt* reached its maximum at 1 h post-dose (3.32-fold of the pre-ENR administration levels). *GYS1* expression showed a sustained decline, reaching its lowest level at 72 h post-ENR administration.

## 3. Discussion

This study represents the first investigation into the pharmacokinetics and tissue residues of ENR in *R. dybowskii*. Additionally, we simultaneously assessed the potential effects of ENR administration on oxidative stress, immune function, and metabolism following its administration. T_max_ is a critical parameter for evaluating the rate of drug absorption within an organism. In this study, T_max_ was observed to be 1 h (in serum), 4 h (in liver), and 8 h (in kidney), respectively, indicating that serum exhibits the fastest absorption rate of ENR among *R. dybowskii* tissues. Similarly, after administering ENR at the same dosage (10 mg/kg) in *Xenopus laevis* serum, T_max_ also occurred at 1 h [20]. This finding suggests that different administration methods may have a minimal impact on the absorption and distribution efficiency of ENR in frog serum. The T_1/2λz_ of ENR in *R. dybowskii* was 12.76 h, which was considerably lower than those determined in largemouth bass (*Micropterus salmoides*) [21], crucian carp (*Carassius auratus gibelio*) [22], and freshwater crocodiles (*Crocodylus siamensis*) [23]. CIP was detected in all tested tissues of *R. dybowskii* following oral ENR administration. However, the CIP levels were much lower than ENR, indicating low ENR conversion in *R. dybowskii*, with most remaining as the parent drug [24]. The ENR and CIP AUC_0−t_ values ranked liver > kidney > serum. The T_max_ was higher than that of the liver, likely attributed to the strong lipophilicity of CIP, which enhances the accumulation capacity of the high-phospholipid kidney for CIP [25]. Chan et al. reported that ENR could convert 10–40% of the C_max_ to its active metabolite CIP depending on the species [26]. In our investigation, the C_max_ ratio of CIP to ENR was 11% in serum. And the AUC_0−t_ ratio of CIP and ENR in *R. dybowskii* serum was 15.5%, which was higher than 3.03% in catfish [27]. This result suggested that, compared to terrestrial animals with AUC ratios ranging from 35% to 55% [23,28], aquatic animals may have a lower degree of ENR metabolic conversion. It is speculated that due to the special habitat, the ENR metabolic capacity of amphibians is stronger than aquatic animals and similar to terrestrial animals. Enrofloxacin in *R. dybowskii* is characterized by rapid absorption and wide distribution. The withdrawal time (WT) of ENR ranged from 12 days to 63 days in many aquatic animals [14,29]. In this study, the WTs were 33 and 34 days in female and male frogs, respectively. The WT of ENR was calculated to be 63 days in rainbow trout (*Oncorhynchus mykiss*) after oral administration via medicated feed at a dose of 10 mg/kg, which was 29 days longer than in Dybowski’s frog. This may be attributed to the low temperature reducing the metabolic rate and elimination rate of ENR [30,31]. Similarly to the *Xenopus laevis* [32], *R. dybowskii* showed a good tolerance of ENR at a dose of 10 mg/kg, but the physiological changes were unknown. Current antibiotic withdrawal period guidelines rely primarily on pharmacokinetic data, overlooking physiological stress recovery in organisms. Therefore, in addition to calculating the withdrawal period, we also studied the physiological changes in two organs, the liver and the intestine.

Studies confirm that fluoroquinolone antibiotics may induce oxidative stress; thus, we measured the related enzyme activities [33]. Therefore, it is speculated that the increase in SOD and CAT activities in this study may be related to the metabolism of ENR in *R. dybowskii*. SOD and CAT served as marker enzymes crucial for scavenging excess ROS, maintaining redox homeostasis, and protecting against oxidative damage [34]. The results are consistent with the previous study, which demonstrated that the activities of SOD and CAT in Chinese soft-shelled turtle (*Pelodiscus sinensis*) were elevated by florfenicol [35]. The levels of malondialdehyde (MDA) were increased after administration in the liver and intestine in *R. dybowskii,* just as in Chinese soft-shelled turtle exposed to ENR [36] and largemouth bass exposed to Oxytetracycline (OTC) [37]. In order to evaluate the potential impact of ENR on oxidative stress in *R. dybowskii*, we further investigated the gene expression of the Keap1/Nrf2 pathway. As the main regulator of antioxidant response, the Keap1/Nrf2 system protects cellular proteins and DNA from oxidative damage caused by reactive oxygen species and electrophiles [38]. Under oxidative stress, Nrf2 triggers the regulation of antioxidant proteins through a signaling cascade [39]. The downstream regulatory genes of the Keap1/Nrf2 pathway include *GST*, *CAT*, and *SOD1*. In our study, 10 mg/kg of ENR reduced the mRNA level of *Keap1* and increased the mRNA levels of *GST*, *CAT*, and *SOD1* in the early stage of administration. As the time of administration increased, the expression levels of *CAT* and *SOD1* decreased. The asynchronous changes in the gene expressions and enzyme activities of SOD and CAT may be caused by the time difference between replication or transcription and translation [40]. Wang et al. discovered that the mRNA levels of *SOD1*, *Keap1,* and *CAT* in loach were also increased after exposure to MPs [41]. To sum up, after ENR administration, *SOD1* and *CAT* scavengers’ free radicals are generated by drug entry into the body, and their expression levels are up-regulated to protect the body from oxidative damage. When the body adapts to the new environment, the expression levels return to normal again. In addition to studying the antioxidant system, we also investigated the effects of ENR on the immune system of *R. dybowskii*. AKP and ACP serve as well-established markers of the innate immune system [42], which are important components of the non-specific immune system. ENR administration could enhance ACP activity and AKP in *R. dybowskii*. In our study, the mRNA level of *IL-1β* was up-regulated 2.3 times at 24 h in the liver. Similar results were obtained in previous studies on largemouth bass [43] and yellow catfish (*Pelteobagrus fulvidraco*) following their exposure to ENR [44]. As a result, the administration of 10 mg/kg of ENR increased the activities of immune-related enzymes (AKP and ACP).

Metabolism encompasses the biochemical reactions occurring within living cells to sustain life [45]. The results indicated that the activities of HK, PK, and PFK were elevated following the administration of ENR, suggesting an enhancement in glycogenolysis. Glycolysis is one of the primary pathways for ATP synthesis, and blood glucose levels may fluctuate when frogs experience conditions of high energy demand. In this study, the significant increase in blood glucose levels can be attributed to heightened metabolic demands induced by ENR treatment. The expression profiles of key genes involved in these metabolic pathways were further analyzed using the quantitative polymerase chain reaction (qPCR). The findings demonstrated that a dosage of 10 mg/kg of ENR resulted in increased mRNA levels of *PI3K*, *Akt*, *IRS4*, and *IGF* while decreasing the mRNA levels of *GYS1*. Consistent with previous research, ENR administration activated the PI3K/Akt signaling pathway in crucian carp (*Carassius auratus* var. Pengze) [46]. In this investigation, the activation of glycolysis was observed in *R. dybowskii* following the oral administration of ENR, which serves to fulfill the energy requirements of these animals. Glucose plays a crucial role as a component of the secondary stress response and exerts significant regulatory effects on energy metabolism and allocation [47]. The enhanced enzymatic activity related to glycogenolysis suggests that frogs require additional energy to expedite the utilization of blood glucose. Given the lack of prior studies examining the relationship between ENR administration and gene expression within the PI3K/Akt pathway, further research is necessary to validate and elucidate our findings. Consequently, gene expression analysis revealed its promotion not only for glycolytic pathways but also for activating both Keap1/Nrf2 and PI3K-Akt signaling pathways.

## 4. Materials and Methods

### 4.1. Chemicals and Reagents

Standard ENR (purity > 98.0%) and CIP (purity > 98.0%) was purchased from Shanghai yuanye Bio-Technology Co., Ltd. (Shanghai, China). ENR powder (purity ≥ 99.0%) used for oral administration was purchased from Beijing Solar bio–Ltd Science & Technology Co. (Beijing, China). The ultrapure water used in this study was prepared with a Veo Lia unit (Veo Lia, Paris, France). Acetonitrile and formic acid were of HPLC grade and purchased from Merck KGaA (Darmstadt, Germany). The corresponding standard was dissolved in sodium hydroxide solution and prepared into a standard solution at a concentration of 1 mg/mL, and the prepared standard solution was diluted with methanol to obtain the standard working solutions of 10 μg/mL and 1 μg/mL.

### 4.2. Animals

This study was undertaken following the ethics requirements and authorized by the Heilongjiang River Fisheries Research Institute (approval number: CAFS20230824-001). A total of 120 (60 males and 60 females) healthy adult Dybowski’s frogs were obtained from Yichun, with an average weight of 15.5 ± 0.1 g. Adult *Rana dybowskii* (30 per tank) were housed in four aquatic tanks. Over a 7-day acclimation period, they were fed drug-free mealworms twice daily at 8:00 AM and 6:00 PM. In these four aquariums, considering the amphibious habitat, we modeled the natural growth environment of Dybowski’s frogs to create an artificial micro-ecosystem. The specific environment is as follows: during the feeding period, water injection and water change were used to keep the water level in the aquarium at about 5 cm, the Dybowski’s frogs perched on the sandbag at about 10 cm, the broad-leaved plants were placed in the tank, and the temperature of the entire micro-ecosystem was 24.0 ± 1.0 °C. Before administration, the experimental frogs were starved for 24 h. Throughout the experiment, no frogs died; they exhibited normal feeding behavior, had intact skin, and showed avoidance responses when approached by humans, indicating their good condition.

### 4.3. Experimental Design

Before ENR administration, 6 frogs (3 females and 3 males) were randomly selected as the non-treated control group (without ENR administration). A suspension with a final concentration of 1.5 mg/mL was prepared by suspending 0.15 g of ENR in 100 mL of sterile water. Before each application, the suspension had to be completely and thoroughly blended. A single oral gavage of 100 μL was administered at each time point, yielding a final dose of 10 mg/kg of body weight. Following the oral gavage of ENR (10 mg/kg), blood, liver, and kidney tissues were collected at 0.25, 0.5, 1, 1.5, 2, 4, 6, 8, 12, 24, 36, 48, and 72 h (n = 6) for pharmacokinetic analysis. Muscle and oviduct tissues were additionally sampled at 1, 3, 7, 15, and 30 days post-dose (n = 6) for drug content determination. Collected blood samples were centrifuged at 1500× *g* for 5 min, and the supernatant was transferred to a new tube for storage as serum.

### 4.4. Sample Preparation and Instrument Analysis

ENR and CIP in serum were detected using detection kits purchased from Shenzhen Finder Biotech Co., Ltd. (Shenzhen, China). According to the instruction manual, 5 μL of serum was mixed with 145 μL of complex solution, and 50 μL was taken for analysis after 1 min of oscillation. The LC-MS/MS method followed the procedure previously described by Liming Chang et al. and was slightly modified [48]. Sample preparation for liver, kidney, muscle, and oviduct tissues followed previously described methods [49,50].

### 4.5. Pharmacokinetic and Residue Analysis

Non-compartmental modeling analyzed ENR and CIP concentration–time data in Dybowski’s frog by WinNonlin 6.1 software. We applied a natural logarithmic transformation to the x-axis to ensure the drug concentration–time curve better aligned with the pharmacokinetic characteristics of antibiotics. The PK parameters were calculated from average ENR or CIP concentrations at each time point, such as peak time (T_max_), peak concentration (C_max_), area under the curve from time zero to infinity (AUC_0−t_), mean residence time (MRT), terminal elimination half-life (T_1/2λz_), and oral clearance (CL/F). The withdrawal time (WT) was calculated by the linear regression analysis of ENR and CIP concentrations in muscle and fallopian tubes in females and muscle in male frogs using the software of WT 1.4 by EMA [51].

### 4.6. Biochemical Assays and Quantitative Real-Time PCR Analysis

Catalase (CAT), superoxide dismutase (SOD), malondialdehyde (MDA), acid phosphatase (ACP), alkaline phosphatase (AKP), hexokinase (HK), pyruvate kinase (PK), phosphor fructokinase (PFK), and aspartate aminotransferase (AST) were detected by a special commercial analytical kit (Suzhou Michy Biomedical Technology Co., Ltd., Suzhou, China). Livers and intestines were prepared following the kit instructions, wherein 0.1 g of tissues was homogenized, and the supernatant was extracted for enzyme activity assays. CAT, SOD, ACP, AKP, HK, PK, PFK, and AST activities were expressed as U/g, and MDA content was expressed as nmol/g.

The total RNA of liver and intestine was isolated using Trizol reagent following the manufacturer’s instructions, and cDNAs were synthesized by a reverse transcription kit (Takara Biotechnology (Dalian) Co., Ltd., Dalian, China). The relative expression levels of GST, PI3K, IL1β, IRS4, JNK1, Keap1, Akt, IGF, AP-1, SOD1, CAT, and GYS1 in liver and intestine were detected, and the primer information is listed in Table 2. The qPCR was performed using a Quant Studio™ 6 Flex instrument (Applied Biosystems, Carlsbad, CA, USA) to quantify the mRNA expression levels. The qPCR reaction conditions followed the methods previously established by our research group [52]. The gene relative expression levels were calculated via the 2^−△△Ct^ method.

### 4.7. Statistical Analysis

ENR concentration–time curves were presented as mean ± standard deviation (SD), while enzyme activity and gene expression data were shown as mean ± standard error of the mean (SEM). A post hoc Tukey–Kramer test was performed using SPSS (SPSS version 26, IBM, Armonk, NY, USA) to analyze the physiological effects of enrofloxacin on *Rana dybowskii*. Significant differences (*p* < 0.05) between groups were denoted by different lowercase letters. Data visualization was conducted using GraphPad Prism 9.4.1.

## 5. Conclusions

In conclusion, our study was the first to explore the PK parameters of ENR and its metabolite, CIP, in *R. dybowskii,* following a single administration of 10 mg/kg. Enrofloxacin in *R. dybowskii* is characterized by rapid absorption and wide distribution. To safeguard frog consumption safety, our findings suggest the maximum WTs for ENR in female and male frogs are 33 and 34 days, respectively. In addition, this study estimated the physiological effect of oral ENR administration in *R. dybowskii*. The administration of 10 mg/kg of ENR increased the activities of glycolytic enzymes (HK, PK, PFK) and immune-related enzymes (AKP and ACP). Gene expression analysis further revealed its promotion of the glycolytic pathway and its activation of the Keap1/Nrf2 and PI3K-Akt signaling pathways. Our study systematically provides pharmacokinetic data for ENR following oral administration in *Rana dybowskii*, thereby offering experimental evidence for rational clinical drug use in this species.

## Figures and Tables

**Table 1:** Pharmacokinetic parameters of ENR in the serum, liver, and kidney.

		ENR			CIP		
Parameters	Unit	Serum	Liver	Kidney	Serum	Liver	Kidney
C_max_	μg/mL (or μg/g)	11.51	11.28	9.11	1.31	4.07	1.39
T_max_	h	1	4	8	1	6	4
AUC_0−t_	(μg/mL) ⋅h or μg/g ⋅h	65.04	142.81	141.21	10.10	32.69	18.74
MRT	h	14.71	17.02	20.04	15.03	14.14	21.97
T_1/2λz_	h	12.76	16.77	17.90	28.42	13.18	81.79
CL/F	mL/h/kg	0.15	0.07	0.06	0.90	0.29	0.32

**Table 2:** Primers for quantitative real-time PCR.

Gene Name	Forward Primer (5′→3′)	Reserve Primer (5′→3′)	Accession Number
*GST*	GCTGATACGGCGCTATCAC	TCTCTCAGAGCCGGTCATGT	XM_040361832.1
*PI3K*	TGGAATCAACTGCCGAGCC	CTGGTGACAGGGTTAAGGG	XM_040349408.1
*IL1β*	ACGTTTGAGTGCCTGTTTGATG	CACTGGTACGGTTGTTCCCT	XM_040345988.1
*IRS4*	CTTCAAGGAGGTCTGCAGG	CTTGTGGACAGGCAAAGGC	XM_040323899.1
*JNK1*	GGTCTGATCCCAGCACATT	TAGCATTGCCATGAGCCCA	XM_040333563.1
*Keap1*	GCGCACTAGGAGTCATGTT	GCTGTTTGTCCGGTGGTTTC	XM_040346421.1
*Akt*	GCTCTCCGAGCGTAACTCC	CGCGGTTTTCAGTCAGTGTG	XM_040332369.1
*IGF*	GATGTACTGTGCTCCTGCCA	TGCACTTCCTTCTGGGCTTT	XM_040344284.1
*AP-1*	ACCGCCTTCACTTTCACAA	TTCGAGTTTCCTCTTCCGGC	XM_040362001.1
*SOD1*	AGGCATGTTGGAGACTTGG	ACTGCTGTGCGTCCAATGAT	XM_040338866.1
*CAT*	TGACTGGCATAACACCCCC	TACCAGGTCCGAAAACAGG	XM_040328095.1
*GYS1*	ACGACCGGGAAGCAAATGAT	ATTCGTGAAGTGAGCCAGG	XM_040325737.1

## Data Availability

Data are contained within the article.
